# 18F-FDOPA PET/CT SUV-Derived Indices and Volumetric Parameters Correlation in Patients with Primary Brain Tumors

**DOI:** 10.3390/cancers13174315

**Published:** 2021-08-26

**Authors:** Agostino Chiaravalloti, Maria Ricci, Andrea Cimini, Francesca Russo, Francesco Ursini, Luca Filippi, Orazio Schillaci

**Affiliations:** 1 Department of Biomedicine and Prevention, University of Rome Tor Vergata, 00133 Rome, Italy; agostino.chiaravalloti@uniroma2.it (A.C.); andreacimini86@yahoo.it (A.C.); orazio.schillaci@uniroma2.it (O.S.); 2 Nuclear Medicine Section, IRCCS Neuromed, 86077 Pozzilli, Italy; 3 UOC Nuclear Medicine, Policlinico Tor Vergata, 00133 Rome, Italy; francescarusso.91@outlook.it; 4 IRCCS Istituto Ortopedico Rizzoli, 40136 Bologna, Italy; francesco.ursini@ior.it; 5 Department of Biomedical and Neuromotor Sciences, University of Bologna, 40127 Bologna, Italy; 6 UOC Nuclear Medicine, Santa Maria Goretti Hospital, 04100 Latina, Italy; lucfil@hotmail.com

**Keywords:** primary brain tumor, PET indices, 18F-DOPA imaging

## Abstract

**Simple Summary:**

This paper aims to improve the knowledge regarding 18F-FDOPA PET/CT parameters that may influence both the interpretation of PET data and the management of primary brain tumors. The evaluation of volumetric parameters in 18F-FDOPA imaging is uncommon, and we aim to increase the scientific interest on the potential role of volumetric parameters in the clinical practice. The standardized uptake value (SUV)-derived indices as SUV max, SUV mean, SUV max ratio, and SUV mean ratio are widely used but the exact methodology to elaborate SUV ratio is not well established. Therefore, this study aims to assess the correlation between SUV-derived indices and volumetric uptake parameters.

**Abstract:**

Novel parameters in PET imaging, such as volumetric parameters, are gaining interest in the scientific literature, but the role of dopaminergic tumor volume (DTV) and total lesion F-DOPA activity (TLDA) and the correlation between volumetric and SUV-derived parameters are not well defined yet. One hundred and thirty-three patients that underwent 18F-FDOPA imaging for primary brain tumors were included in this retrospective study. SUV-derived indices were calculated (the occipital region was chosen to generate ratios of tumor SUV) and compared with volumetric parameters. Regression models were applied in univariate analysis and lnSUVmax was positively associated with lnDTV (beta 0.42, *p* = 0.007), the lnSUVmax ratio was positively associated with lnDTV (beta 0.80, *p* = 0.011), lnSUVmax was positively associated with lnTLDA (beta 1.27, *p* < 0.0001), and the lnSUVmax ratio was positively associated with lnTLDA (beta 1.87, *p* < 0.0001). Our study demonstrates that volumetric uptake parameters in 18F-FDOPA PET/CT are easier to assess in primary brain tumors with higher SUV max and SUV max ratios, and supports the emerging role of volumetric parameters in the data interpretation.

## 1. Introduction

Positron emission tomography (PET) with the amino-acid analog 3,4-dihydroxy-6-18 F-fluoro-l-phenylalanine (18 F-FDOPA) is one of the most used imaging techniques in the management of primary brain tumors, especially for tumor grading, delineation of tumor extension, treatment planning, treatment response, and post-treatment surveillance [1]. This radiopharmaceutical is a catecholaminergic precursor, and it enters the cell through L-type amino acid transporter 1 and 2 (LAT1 and LAT2), which are overexpressed in tumor cells [2]. Inside the cell, 18F-FDOPA is decarboxylated by aromatic l-amino acid decarboxylase with the formation of 18F-dopamine, and is then transported into vesicles [3]; the increased protein synthesis induces a high radiopharmaceutical uptake in tumor cells.

The recent World Health Organization (WHO) Classification of Tumors of the Central Nervous System (2016) describes primary brain tumors based on histopathologic features and molecular genetic alterations [4]. The possible effects of these characteristics on 18 F-FDOPA uptake in primary brain tumors (PBT) have been studied: for instance, the correlation between 18 F-FDOPA uptake in brain tumor cells and glioma grade has been analyzed by several authors [5,6], with higher uptake of the radiotracer in high-grade gliomas; moreover, the possible effects of molecular-genetic alterations of gliomas on amino-acid metabolism using 18 F-FDOPA PET/computed tomography (CT) has been investigated, showing no significant correlations in the two studies [7,8].

The use of standardized uptake value (SUV) is common in clinical practice for assessing the uptake of radiopharmaceuticals by tumor lesions in PET/CT scans: SUV is calculated as the ratio of tissue radioactivity concentration at a given time, divided by the dose at the time of injection divided by body weight [9]. Moreover, tumoral uptake is usually evaluated with the SUV-derived indices as SUV max, SUV mean, SUV max ratio, and SUV mean ratio. 18F-FDOPA PET SUV-derived indices are routinely available information that also enables an accurate discrimination of low-grade and high-grade gliomas [10]. However, the exact methodology to elaborate SUV ratio is not well established; mainly, the background region selected to generate ratios of tumor uptake is not all-encompassing.

This paper aims to focus on the SUV max ratio calculation methodology. Since none of the patients examined had PBT in the occipital regions as detectable by both PET/CT and MRI data, this site has been selected for SUVmax calculation for the background, based on previous studies of our research group [8,11,12] in which the occipital region was chosen in order to generate ratios of tumor SUV.

However, novel parameters in PET imaging, such as volumetric parameters, are gaining interest in scientific literature. Several studies, reviews, and metanalysis about 18FDG-PET/CT focused on the growing role of volumetric parameters in several categories of cancers [13,14]. Most of the published papers regarding the prognostic value of the volumetric parameters are related to nasopharyngeal carcinoma [14], and several papers focused on the thoracic region (non-small cell lung cancer, breast cancer, esophageal cancer) [15,16,17] and other carcinomas, supporting the role of volumetric parameters in 18F-FDG imaging, especially in the prognostic phase.

However, the role of volumetric 18F-FDG PET parameters in brain tumors is still a matter of debate.

In the field of neuro-oncology, the volumetric parameters have also been studied in 18F-FET PET/MRI imaging in a recent paper that compared the 18F-FET-derived tumor volume, VFET, with tumor volume depicted by using MRI findings. This resulted in findings that were not always congruent between FET PET and MRI, which may reflect different metabolic properties of gliomas [18]. Nevertheless, a further study compared the tumor volume elaborated by considering the uptake of 18F-FET and the CT alone, supporting the role of the 18F-FET PET imaging for RT planning and volume delineation, especially in patients that present contraindications to MRI [19]. In addition, a recent meta-analysis of 11C-methionine-PET suggests a correlation between volumetric parameters and prognostic data as event-free survival and overall survival, underling the importance of volumetric parameters in 11C-methionine PET imaging in the prognosis phase [20].

However, the evaluation of volumetric parameters in 18F-FDOPA imaging is not typical, and, to our knowledge, few studies have been published. The calculation methods of volumetric parameters are similar in 18F-FDG and 18F-FDOPA imaging, but the outputs are different: dopaminergic tumor volume instead of metabolic tumour volume, total lesion F-DOPA activity instead of total lesion glycolysis. A dedicated tool in the workstation allows the calculation of volumetric parameters based on a re-elaboration of the activity detected in the VOI elaborated by the nuclear medicine physician: as described in a previous paper [21] in primary brain tumors, 40% of SUVmax was used to generate volume of interest. If the 40% of SUVmax was below 2.5, an SUV threshold of 2.5 was used instead. However, similarly to volumetric parameters in 18F-FDG PET/CT imaging, the volumetric parameters in 18F-FDOPA imaging may support the prognostic phase [21]. Moreover, to the best of our knowledge, in evaluating primary brain tumors using 18F-FDOPA PET/CT, the correlation between SUV-derived indices and volumetric uptake parameters such as dopaminergic tumor volume (DTV) and total lesion F-DOPA activity (TLDA) is not well defined yet.

This study aims to assess the correlation between SUV-derived indices and volumetric uptake parameters in 18F-DOPA PET/CT scans of patients with primary brain tumors, in order to improve the knowledge regarding parameters that may influence the management and the image analysis of primary brain tumors.

## 2. Materials and Methods

### 2.1. Patients’ Selection

One hundred and thirty-three patients with PBT were recruited in this retrospective paper (61 women and 72 men; mean age 46.1 ± 14.1 years old). All involved in the study underwent PET/CT examination with 18F-FDOPA either at Policlinico Tor Vergata (Rome, Italy) or at Istituto Neurologico Mediterraneo Neuromed (Pozzilli, Italy) between December 2011 and March 2019.

The study was performed according to the Declaration of Helsinki. Considering the double-centric nature of the study, this retrospective analysis has been approved by the local ethics committee of the Tor Vergata University in Rome and by Comitato Etico Istituto Neurologico Mediterraneo Neuromed. All participants or their legal guardians gave the written informed consent for the PET imaging scan, but the study consent form was waived due to the retrospective nature of this observational study. The subjects included in the retrospective analysis were adult (≥18 years old), had a diagnosis of primary brain tumor, and were willing to participate in an eventual future retrospective study (all patients involved in the study agreed to sign a section of the routinely PET imaging consent form, that allows the usage of data for future eventual observational study).

Of the 133 patients:

- 60 patients were affected by astrocytoma (45.1%), of which one patient was affected by Grade I pilocytic astrocytoma, 28 patients were affected by Grade II astrocytoma, and 15 patients were affected by Grade III astrocytoma;
- 4 were affected by Grade III anaplastic astrocytoma (3%);
- 11 were affected by glioma (8.3%) of which 3 patients were affected by Grade II glioma, 1 patient was affected by Grade III glioma, and 1 patient was affected by glioma (grading not identified/not reported);
- 44 were affected by oligodendroglioma (33.1%) of which 12 patients were affected by Grade II oligodendroglioma, and 8 patients were affected by Grade III Oligodendroglioma;
- 6 were affected by by Grade II oligoastrocytoma (4.5%);
- 7 were affected by Grade IV glioblastoma (5.2%);
- 1 was affected by neurocytoma (0.8%).

### 2.2. F-FDOPA PET Image Acquisition, Parameters, and Image Evaluation

According to other similar reports from our research group, PET/CT was performed in the 18F-FDOPA group [8,11,12]. For the reconstruction of PET images, we used ordered subset expectation maximization (OSEM) with standard technique with four iterations and 20 subsets of data were acquired in a 3D model in a 256 × 256 matrix for the acquisition of the images. Before functional imaging acquisition, a low-amperage CT scan of the head for attenuation correction (40 mA; 120 kV) was performed. PET/CT images were acquired using a Discovery VCT or a Discovery ST 16 scanner (GE Healthcare, Chicago, IL, USA) 20 min after the radiolabeled compound injection, with a scanning time of 30 min. Patients fasted for at least 4 h before 18F-DOPA administration. All participants were injected intravenously with a radiolabeled compound of 18F-DOPA, with the mean activity of 4 MBq/kg (185 ± 75 MBq). In addition, all subjects were hydrated with 500 mL of NaCl 0.9%. Carbidopa was not administered before radiopharmaceutical injection.

A volume of interest (VOI) on the tumor site was traced by an experienced nuclear medicine physician, starting from the slice with the highest uptake of the radiopharmaceutical, with the support of co-registered MRI images by using SPM8 software (thus permitting a correct placement of the VOI, even in lesions with striatal involvement).

From the placed VOI, the maximum standardized uptake value for the site of recurrence (SUVmax lesion, defined as the hottest voxel in the VOI) and the mean standardized uptake value for the site of recurrence (SUVmean lesion, the average SUV of voxels in the VOI) was calculated on a dedicated workstation (version 4.4, Advantage Workstation, GE Healthcare, Chicago, IL, USA).

According to previous studies of our research group [8,11,12], the occipital region was chosen for SUVmax calculation for the background (SUVmax occ), obtained using a standard VOI of 1.5 cm × 1.5 cm ×1.5 cm, placed on the occipital lobe. The occipital region, that was unaffected in all subjects involved as detectable by both PET/CT and MRI data, has been chosen as background instead of the striatum or the contralateral hemisphere in order to avoid discrepancies due the eventual affection of these cortical regions [8,11,12].

SUVmax ratio was calculated as SUVmax lesion/SUVmax occ, as proposed by Chiaravalloti et al. in a previous report [12].

**Figure 1:**
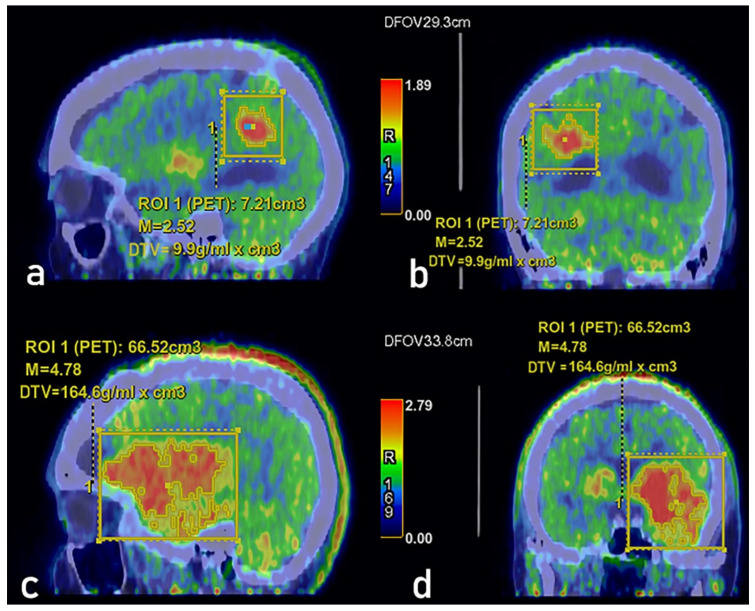
Dopaminergic tumor volume (indicated as DTV in the figure) calculation on a dedicated workstation (version 4.4, Advantage Workstation, GE Healthcare, Chicago, IL, USA) in a patient with Grade II glioma in sagittal (**a**) and coronal (**b**) views, and in a patient with Grade IV glioblastoma in sagittal (**c**) and coronal (**d**) views. The values of DTV are correlated to SUV max (see the text).

DTV was recorded as F-DOPA volumetric parameters (unit in cubic centimeter) on a dedicated workstation (version 4.4, Advantage Workstation, GE Healthcare, Chicago, IL, USA) and TLDA were calculated as the SUVmean of the volume of interest multiplied by DTV, following the nomenclature and the approach proposed by Liu et al. [21] (Figure 1).

### 2.3. Statistical Analyses

All variables were highly skewed and therefore were ln-transformed before the analysis. Linear regression models were built to evaluate the association between independent variables as lnSUVmax and lnSUVmax ratio, and lnDTV and lnTLDA (dependent variable).

## 3. Results

**Figure 2:**
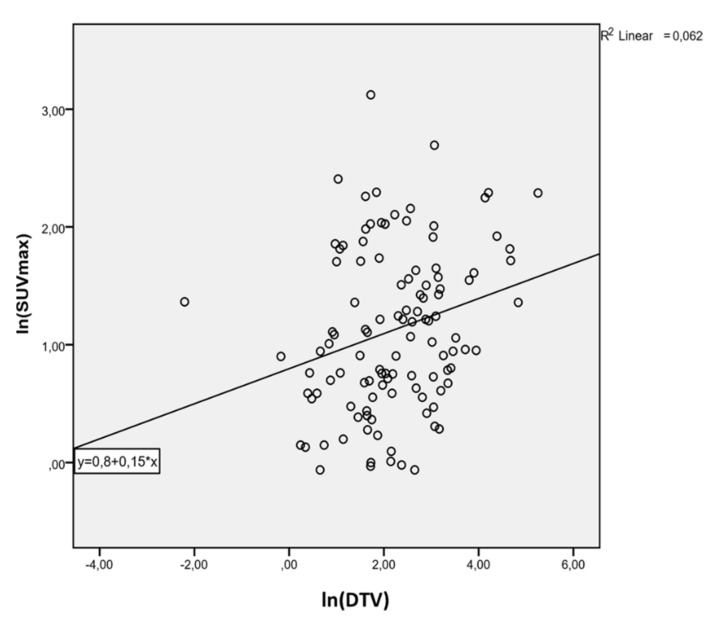
Univariate analysis model of the association between lnSUVmax and lnDTV.

**Figure 3:**
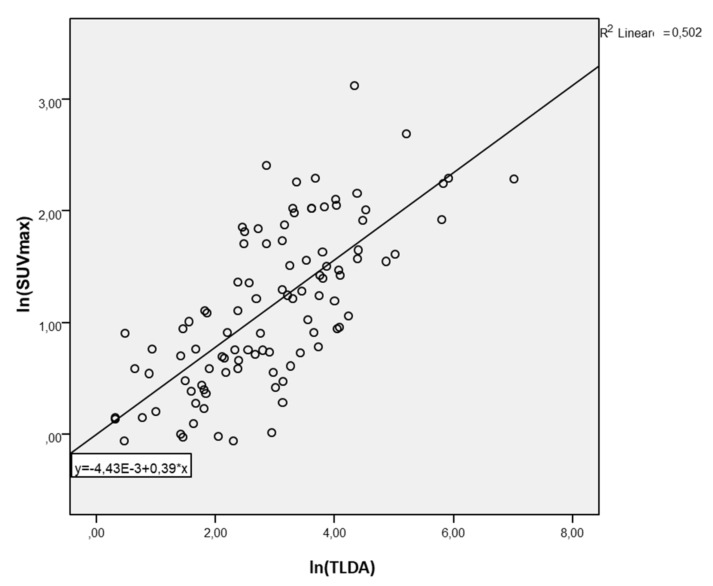
Univariate analysis model of the association between lnSUVmax and lnTLDA.

**Figure 4:**
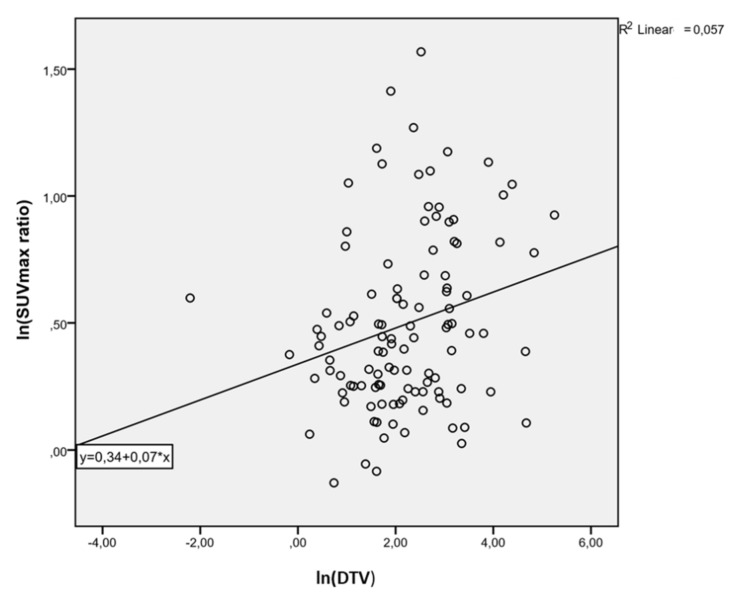
Univariate analysis model of the association between lnSUVmax ratio and lnDTV.

**Figure 5:**
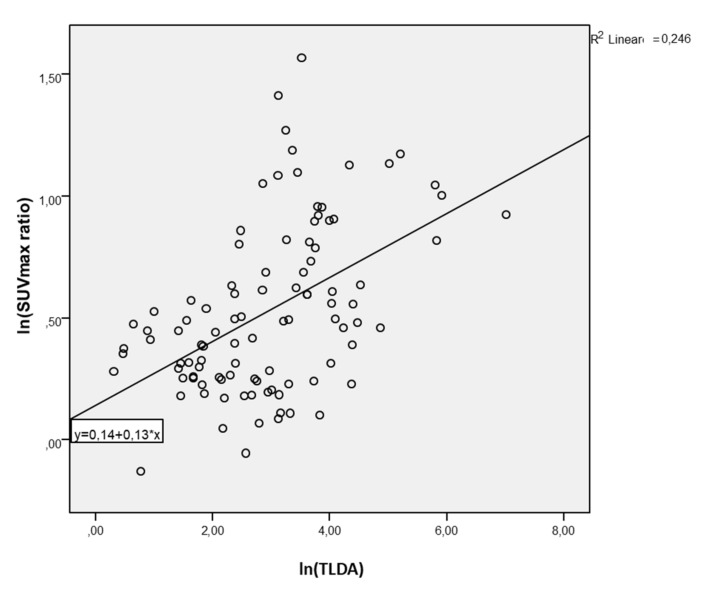
Univariate analysis model of the association between lnSUVmax ratio and lnTLDA.

In univariate analysis, lnSUVmax was positively associated with lnDTV (beta 0.42, *p* = 0.007); the model explained 6.2% of the variance in lnDTV (Rsquare 0.62, StErr 1.13) (Figure 2). Furthermore, the lnSUVmax ratio was positively associated with lnDTV (beta 0.80, *p* = 0.011); the model explained 23.8% of the variance in lnDTV (Rsquare 0.238, STED 1.13) (Figure 3). Similarly, lnSUVmax was positively associated with lnTLDA (beta 1.27, *p* < 0.0001); the model explained 50.2% of the variance in lnDTV (Rsquare 0.502, STED 0.93) (Figure 4). The lnSUVmax ratio was positively associated with lnTLDA (beta 1.87, *p* < 0.0001); the model explained 24.6% of the variance in lnDTV (Rsquare 0.246, STED 1.14) (Figure 5).

## 4. Discussion

Primary brain tumors are remarkable for their clinical heterogeneity with a broad spectrum of tumor behaviors. MRI is currently the reference imaging technique for diagnosis and follow-up of these tumors. T1- and T2-weighted sequences reveal tumoral size and location with high sensitivity. However, MRI has some inconveniences, and therefore, multimodal imaging that includes functional imaging is the most-used approach. New PET tracers such as amino acid tracers are highly informative. For example, the role of amino acid radiopharmaceuticals PET/CT imaging in brain tumor management is well confirmed and widely used in clinical practice, including 18F-FDOPA imaging. In 18F-FDOPA imaging, beyond the visual interpretation, SUV-derived indices as SUV max and SUV max ratio are routinely available information used in the clinical practice that may enable a better interpretation of PET/CT data [10]. In contrast, the correlation of tumour characteristics with PET/CT indices is still to be established, particularly the role of volumetric uptake parameters, as DTV and TLDA. In fact, to our knowledge, even if there are more pieces of evidence concerning the volumetric uptake indices of 18F-FDG imaging, few studies focused on the role of volumetric parameters in 18F-FDOPA imaging [20]. However, due to the promising role of volumetric indices in 18F-FDG imaging, we aim to focus on volumetric indices also in 18F-FDOPA imaging. Therefore, in addition to SUVmax and SUVmax ratio, the volumetric uptake parameter values were also discussed in this study. The aim of our study is to evaluate the correlation between volumetric parameters and SUV-derived parameters, in order to improve the knowledge concerning the PET/CT indices that may influence brain tumor management, especially the prognostic aspects according to the previous data available.

In a previous report, both DTV and TLDA in 18F-DOPA imaging correlated more highly with risk grouping in pediatric population with neuroblastoma that underwent a pre-treatment imaging with both 18F-FDOPA and 18F-FDG [21]. Authors evaluated both methodologies’ volumetric parameters and all indices compared between groups distinguished by survival status and clinical features: pretherapeutic 18F-DOPA and 18F-FDG PET provided complementary information volumetric indices of 18F-DOPA and 18F-FDG PET correlate more highly with risk grouping. These findings, even if related to a population different from our paper population in terms of age and histopathology, confirm the potential role of volumetric PET/CT indices in 18F-FDOPA. The role of PET/CT indices in tumor management, particularly in the prognostic phase, generates interest, and therefore, we aim to correlate DTV and TLDA with ordinary SUV-derived indices, to confirm the increasing importance of 18F-FDOPA parameters in tumor management with potential implications, particularly in prognostic aspects of 18F-FDOPA data interpretation and, subsequently, in the primary brain tumor management.

Moreover, 18F-FDOPA PET SUV-derived indices are routinely available information easily accessed by using different dedicated workstations. Although patients with glioma recurrence/progression can be detected by static and dynamic 18F-FDOPA PET parameters, most of this diagnostic information can be achieved by conventional static parameters [22] avoiding logistical disadvantages associated with the dynamic acquisition as the increased acquisition time. According to our results, both SUV max and SUV max ratio positively correlate with DTV and TLDA. Therefore, our findings suggest that 18F-FDOPA volumetric parameters, being correlated to uptake value as described by the correlation with SUV-derived indices (both SUV max and SUV max ratio), are associated with an improved and more accessible evaluation in case of a higher uptake of radiopharmaceutical (in case of higher SUV). A previous paper used an SUV of 2.5 as the threshold for MTV and TLDA calculation [21]. In a further paper, the metabolic tumor volume (MTV) was obtained through a 3D auto-contouring process with a threshold corresponding to the SUV mean of the contra-lateral striatum [22]. However, different papers are needed in order to evaluate a specific minimum SUV threshold for the correct evaluation of volumetric PET/CT parameters, but our results confirm this trend.

We also aim to focus on the methodology concerning the calculation of SUV max ratio, based on previous studies of our research group [8,11,12] in which the occipital region was chosen for SUV max calculation for the background (SUVmax occ), instead of using an ROI positioned on the semi-oval center of the unaffected contralateral hemisphere, as reported in other papers [23]. The occipital region was chosen as background region because it was unaffected in all subjects involved in the study (contrarily to the striatum or the contralateral hemisphere that were affected in few cases in our sample) in order to avoid discrepancies in the data analysis.

However, the methodology concerning the SUV ratio calculation is heterogeneous and is still a matter of debate. The exact methodology of the background selection is not clearly defined and, our research group published further papers with the occipital region as background for the SUV ratio calculation, with promising results [8,11,12]. Indeed, the usage of the occipital region is particularly helpful in case of affection of the alternative areas widely used, as striatum or contralateral hemisphere, or in case of further clinical reasons that may affect the metabolism of these regions. Our study is the first one, to our knowledge, that identifies the SUV ratio based on occipital background and that compared SUV max ratio (tumor-to-occipital) with volumetric parameters: the SUV max ratio was positively associated with DTV (*p* = 0.011), and the best correlation was with the SUV max ratio and TLDA (*p* < 0.0001). These results confirm our methodology’s appropriateness concerning the evaluation of the SUV max ratio, and, on the other hand, they give a novel insight into the volumetric 18F-FDOPA indices.

A previous paper associates the SUV max ratio (evaluated by using the contralateral uptake as background) with prognostic factors, supporting the importance of SUV-derived indices in prognostic management, including the grading and the overall survival [6]. In a further paper, the SUV max ratio was calculated considering the striatum uptake (tumor-to-striatum ratio) in low-grade gliomas [24].

However, according to a previous paper, all SUV-derived indices studied (including SUV max) enable accurate discrimination of low-grade and high-grade gliomas, and the best-correlated indices were SUV mean tumor/normal brain ratio (T/N) and SUV mean tumor/striatum ratio (T/S). Therefore, in this paper, the ratios of tumor uptake to normal tissue uptake were generated by dividing tumor SUVs by the SUVs of the contralateral centrum semi-oval (T/N) and by the SUV of the striatum (T/S) [10].

However, the SUV max ratio was also calculated considering that occipital uptake was associated with a good prognostic performance, correlating both to progression-free survival (PFS) and the overall survival (OS) in a population affected by primary brain tumors [11].

Contrarily, in a further study, 18F-FDOPA PET/MRI imaging results for monitoring the response to treatment reported that maximum and mean SUVs, as well as tumor-to-brain ratios, were not predictors of response. On the other hand, in the same paper, authors described a trend towards the percent DTV change seen on the 4-week scan correlating with progression-free survival [25]. In a further paper that determined the prognostic value of volumetric parameters derived from pre-treatment 18F-FDG and 18F-DOPA PET/CT of neuroblastoma and their correlation with clinical and histopathologic features, only volumetric indices (DTV, TLDA, MTV, and TLG) significantly differed among risk groups [21].

It seems clear that the emerging role of volumetric uptake parameters is gaining interest in scientific literature with heterogeneous findings, and therefore, further studies focused on correlation with other variables, including other PET parameters, are needed. In addition, our results confirm the role in clinical practice of the SUV max ratio, by supporting the novel insight in the selection of the occipital region as background region, especially in cases of affection of alternative areas (striatum and contralateral hemisphere) or in case of DOPA uptake alterations in these areas due to different clinical conditions.

Further multicentric trials on larger samples are needed for eventually elaborating an appropriate classification of patients with primary brain tumor based on PET/CT indices, but to date, we can consider PET/CT indices as valuable tools that may help in the image analysis. However, our findings confirm the importance of calculating SUV-derived indices and volumetric uptake parameters in the PET/CT assessment. Remarkably, the best correlation is described between both SUV max and SUV max ratio and TLDA. These results support the gained interest in volumetric 18F-FDOPA indices, especially for TLDA. A limitation of the present paper is the absence of a statistical subgroups analysis based on the histopathological findings. An additional subgroup analysis has not been performed because some samples would not be comparable or randomized due to the different number of patients involved in the study between histopathological subgroups. The correlation between volumetric parameters and histopathological finding should be explored in further studies in larger samples.

In addition, the association between volumetric parameters and specific clinical aspects or prognostic factors should be explored in further papers. In fact, a further limitation of our study is represented by the lack of data regarding prognostic indicators as overall survival and/or progression-free survival.

## 5. Conclusions

Our study supports the growing role of volumetric uptake parameters in 18F-DOPA PET/CT in clinical practice. Our report demonstrated that volumetric parameters in 18F- DOPA imaging are easier to assess in primary brain tumors with a higher SUV max and SUV max ratio. Our results also confirm the important role of the SUV max ratio, calculated in our sample by using tumor-to-occipital ratios, in the image analysis of primary brain tumors with 18F-DOPA PET/CT.

## Data Availability

Not applicable.
